# A randomised cross-over trial in healthy adults indicating improved absorption of omega-3 fatty acids by pre-emulsification

**DOI:** 10.1186/1475-2891-6-4

**Published:** 2007-01-25

**Authors:** Iveta Garaiova, Irina A Guschina, Sue F Plummer, James Tang, Duolao Wang, Nigel T Plummer

**Affiliations:** 1 Obsidian Research Ltd., Baglan, Port Talbot, UK; 2 Cardiff School of Biosciences, Cardiff University, Cardiff, UK; 3 Medical Statistics Unit, London School of Hygiene & Tropical Medicine, London, UK; 4 Cultech Biospeciality Products, Research Department, Unit 3 Christchurch Road, SA12 7BZ Port Talbot, UK

## Abstract

**Background:**

The health benefits of increased intakes of omega-3 fatty acids are well established but palatability often presents a problem. The process of emulsification is used in the food industry to provide a wider spectrum of use, often with the result of increased consumption. Moreover, as emulsification is an important step in the digestion and absorption of fats, the pre-emulsification process may enhance digestion and absorption. In this study the levels of plasma fatty acid and triacylglycerol (TAG) following the ingestion of either an oil mixture or an emulsified oil mixture have been compared.

**Methods:**

In this randomised cross-over study, 13 volunteers received the oil mixture and 11 received the oil emulsion as part of an otherwise fat free meal. Blood samples were collected at 0, 1.5, 3, 4.5, 6, 7.5 and 9 hours after ingestion of oil, separated and stored at -20°C. Plasma triacylglycerols were assessed spectrophotometrically and fatty acids were determined by gas chromatography. Following a washout period of twenty days the procedure was repeated with the assignments reversed.

**Results:**

The postprandial plasma TAG and the C18:3 (n-6), C18:3(n-3), C20:5(n-3) and C22:6 (n-3) polyunsaturated fatty acid (PUFA) levels for the emulsified oil group were increased significantly (P = 0.0182; P = 0.0493; P = 0.0137; P < 0.0001; P = 0.0355 respectively) compared with the non-emulsified oil group. The C16:0 and C18:0 saturated fatty acids, the C18:1 (n-9) monounsaturated fatty acid and the C18:2 PUFA were not significantly different for the oil and emulsified oil groups.

**Conclusion:**

Pre-emulsification of an oil mixture prior to ingestion increases the absorption of longer chain more highly unsaturated fatty acids (especially eicosapentaenoic acid and docosahexaenoic acid) but does not affect absorption of shorter chain less saturated fatty acids, suggesting that pre-emulsification of fish oils may be a useful means of boosting absorption of these beneficial fatty acids.

**Trial registration:** Current Controlled Trials ISRCTN43202606

## Background

The biochemical importance of dietary lipids to human physiology and nutrition is now firmly established and research continues to show potential health benefits of polyunsaturated fatty acids (PUFAs) and in particular the omega-3 fatty acids both from fish and precursors of the same family found in many plant oils [1-3].

Current UK recommendations advise the public to eat at least two portions of fish every week including one of oily fish, which is rich in omega-3 PUFAs, or take >200 mg/day of omega-3 PUFAs [4]. According to the WHO, one portion should provide an equivalent of 200–500 mg of eicosapentaenoic acid (EPA) and docosahexaenoic acid (DHA) [5]. In fact most people in the UK consume considerably less than one portion per week [4] indicating that the recommended levels are not being achieved leaving fish oil supplementation as the most convenient route to augment dietary intake. However, reaching recommended intake levels may require taking several capsules of fish oil per day. An alternative is to use a more palatable version of liquid fish oils presented as a flavoured emulsion which may offer a more flexible route to achieve the required level of intake.

Approximately 95% of dietary lipids ingested from foods consist of triacylglycerols (TAGs) and lipid digestion is initiated by the action of lingual/gastric lipases which together with the physical mixing action of the stomach produces small partially emulsified droplets containing mainly TAGs but also free fatty acids (FFA) and diacylglycerols. Further emulsification together with the action of pancreatic lipase results in the production of 2-monoacylglycerols (sn2-MAG) and free fatty acids and these are incorporated into mixed micelles before passive diffusion into the enterocyte [6,7]. Once inside the enterocyte the fatty acids with less than 12 carbon atoms pass directly into the hepatic portal vein [6]. However, most dietary fatty acids have a chain length greater than 12 and these are re-synthesised into TAG and incorporated into the chylomicron (CM) family of lipoproteins [7], which are secreted into the lymphatic system and transported to the thoracic lymphatic duct where they enter the circulation [8]. The fatty acid composition of the core of the CM – TAG generally reflects the dietary fatty acid profile, particularly in the first 4–6 hours postprandial period [9]. The CM-TAGs are rapidly hydrolysed by lipoprotein lipase (LPL) releasing between 70–90% of the total TAG and leaving chylomicron remnants which are removed from the plasma via low density lipoprotein (LDL) receptors in the liver [10,11].

There have been conflicting observations that the use of emulsified forms of fish oils in some studies has led to improved digestion and absorption of EPA and DHA [12,13] with other work showing no differences in absorption [14]. Preliminary research in our laboratory has indicated that both the rate and extent of absorption of the fatty acids EPA and DHA improve if the oil is pre-emulsified prior to ingestion. This cross – over absorption study was designed to establish whether pre-emulsification of an oil mixture leads to improved absorption of fatty acids, particularly DHA and EPA, compared with the non-emulsified form of the oil mixture.

## Methods

### Subjects

Twenty four healthy volunteers (14 females and 10 males, 5 smokers) were enrolled into the study. The mean age of participants was 35.13 ± 11.59 years and their BMI was 24.66 ± 5.19 kg/m^2^. Volunteers were excluded from the study if they had been involved in dietary control, were receiving long-term medication for the chronic diseases, were obese or had very low body weight, had diagnosed hypercholesterolemia, coronary artery disease or diabetes. Participants who were taking dietary fatty acid supplements were also excluded.

All volunteers provided written informed consent before participating in the present study and ethical permission was obtained from Iechyd Morgannwg Health, Local Research Ethics Committee, United Kingdom.

### Study design and diet

In a cross-over study, participants were randomly assigned to receive either the oil mixture or oil emulsion mixture, with thirteen subjects receiving oil and eleven subjects receiving oil emulsion in the first stage.

The mixtures of oils (comprising: concentrated fish oil 43%, borage oil 31% and flaxseed oil 26%) in both the natural form and in the emulsified form were supplied by Cultech Ltd., Port Talbot, United Kingdom. The fatty acid composition of the oil preparations is shown in Table 1.

**Table 1:** Fatty acid composition of the oil mixture (natural and emulsified forms).

**Fatty acid**	**wt %**
Myristic acid (14:0)	0.15
Palmitic acid (16:0)	3.7
Palmitoleic acid (16:1)	0.48
Stearic acid (18:0)	3.8
Oleic acid (18:1 n-9)	10.4
Vaccenic acid (18:1 n-7)	1.25
Linoleic (18:2 n-6)	9.5
alpha-Linolenic acid [ALA] (18:3 n-3)	18.2
gamma-Linolenic acid [GLA] (18:3 n-6)	8.3
Stearidonic acid (18:4 n-3)	0.75
Eicosadienoic acid (20:2 n-6)	0.25
Arachidonic acid [ARA] (20:4 n-6)	0.9
Tetracosapentaenoic acid (20:4 n-3)	0.75
Eicosapentaenoic acid [EPA] (20:5 n-3)	16.8
Adrenic acid (22:4 n-6)	0.1
Docosapentaenoic acid (22:5 n-3)	2.7
Docosahexaenoic acid [DHA] (22:6 n-3)	11.0
*Others	10.91

*Consist of fatty acids comprising no more than 1.5% of total fatty acid content

The participants fasted for 12 hours prior to the study. Subjects consumed 30 ml of oil as part of a meal comprising fat free cereal (37.5 g), skimmed milk (230 ml), sugar (4 g) and apple juice (150 ml). For the remainder of the 9 hour collection period the participants ate only boiled rice (100 g), boiled pasta (100 g) and fresh fruit (large apple). Apple juice or water was available to drink throughout the day. The average energy intake was 3596 kJ (859 kcal) comprising 147 g carbohydrate, 23 g protein and 32 g total fat. Twenty days later (washout period), the procedure was repeated with the assignments reversed.

Of the 24 subjects recruited, 1 female subject was withdrawn being unable to provide sufficient volume of blood and a second female only provided sufficient volume for triacylglycerol analysis.

### Blood specimen collections

To monitor progression of the digestion and absorption of TAG and individual fatty acids over the postprandial period, an initial fasted venous blood sample (5 ml) was collected from each volunteer into heparin tubes followed by samples at 1.5, 3, 4.5, 6, 7.5 and 9 hour after ingestion of oil/emulsion. Plasma samples were obtained by centrifugation at 1000 *g *for 15 min at 4°C and stored at -20°C until analysis at Cardiff School of Biosciences.

### Lipid extraction from plasma and analysis of triacylglycerols

Lipids were extracted from 0.5 mL of plasma with chloroform:methanol (1:2, v/v) by the method of Garbus et al. [15]. Triacylglycerols were separated by thin-layer chromatography with a mixture of hexane: diethyl ester: acetic acid (90:30:1, v/v/v) as the elution phase and quantified by determination of ester bonds [16].

### Analysis of total fatty acids from plasma

Fatty acid methyl esters (FAME) were prepared by incubation for 2 h with 2.5% H_2_SO_4 _in dry methanol at 70°C. FAME were analysed by gas chromatography using an Agilent Technologies 6890 N gas chromatograph, fitted with a 7683 series injector and a Varian FactorFour™ (VF-5 ms; 30 m × 0.25 mm i.d., 0.25 μm film thickness; Varian Limited UK, Oxford, UK). The column temperature was held at 170°C for 3 min, then temperature-programmed to 220°C at 4°C/min. Helium was the carrier gas at a flow rate 2 ml/min. The fatty acid esters were identified by comparison of their retention times with authentic standards and pentadecanoate (C15:0) was used as an internal standard. Fatty acid standards were obtained from Nu-Chek Prep. Inc. (Elysian, MN, USA).

### Statistical analysis

For all parameters, the area under the concentration curve (AUC) was calculated by subject and period by means of the trapezoidal rule after correction for the baseline concentration of lipids. AUC represents the integration of absorption and clearance of a triacylglycerol or fatty acid. The AUCs were then analysed by an ANOVA model with repeated measurements taking into account the cross-over design. In the ANOVA model, Treatment (oil or emulsion), Period (Day 0 and Day 20) and Sequence (oil-emulsion or emulsion-oil) were introduced as fixed effects and subject as a random effect. Reported P-values are two-sided, and all statistical analyses were carried out by using the Statistical Analysis System (SAS) version 9.1 (SAS Institute, Inc., Cary, NC, USA). There was no statistically significant interaction between Sequence and Treatment or between Period and Treatment.

## Results

Analysis of the baseline plasma concentrations of total TAG and individual fatty acids for the oil and the emulsion groups at Day 0 and Day 20 indicated that these two populations were comparable (Table 2). It was also found that the two treatment sequences were similar (results not shown).

**Table 2:** Comparison of the baseline plasma concentrations of total TAG and individual fatty acids.

**Lipid Component**	**Mean plasma concentration at Day 0**	**Mean plasma concentration at Day 20**
Palmitic acid (16:0)	603.1 ± 215.5	617.9 ± 173.8
Stearic acid (18:0)	168.8 ± 53.6	179.1 ± 45.9
Oleic acid (18:1 n-9)	536.0 ± 186.9	564.2 ± 142.4
Linoleic acid (18:2 n-6)	780.5 ± 281.8	816.0 ± 239.3
α Linolenic acid (18:3 n-3)	16.7 ± 8.1	17.1 ± 6.4
γ Linolenic acid (18:3 n-6)	12.0 ± 7.7	11.6 ± 7.4
Eicosapentaenoic acid (20:5 n-3)	25.7 ± 17.5	33.9 ± 21.8
Docosahexaenoic acid (22:6 n-3)	61.4 ± 26.9	73.0 ± 34.6
Triacylglycerols	536.8 ± 182.5	533.7 ± 157.4

Fatty acid data are expressed as mean (mg/l) ± SD for Day 0 and Day 20 (n = 22). Triacylglycerol data are expressed as mean (μmol/l) ± SD for Day 0 and Day 20 (n = 23).

SD – standard deviation; n – number of subjects

**Figure 1:**
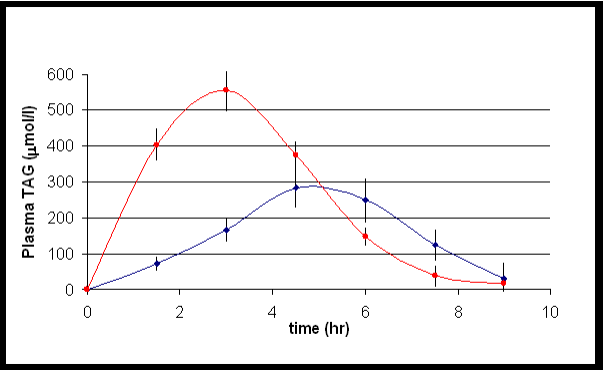
**Comparison of the concentration of plasma triacylglycerols for oil and emulsion groups**. Values represent Mean ± SEM of individuals (n = 23) at each time point. -◆- (blue) oil group; -▪- (red) emulsion group; SEM – standard error of the mean; n – number of subjects.

The rate and total extent of TAG absorption for the two treatment groups is illustrated in Figure 1 and it can be seen that both were greater for the emulsion group than the oil group with a significantly lower total absorption of TAG for the oil group (AUC difference between oil and emulsion groups: 904.20 hr. μmol/l) with the ratio between the emulsion and oil group being 60.4% (P = 0.0182; 95% CI of 169.9 to 1638.5).

The difference seen in plasma TAG levels between the oil and emulsion groups is explained largely by an improved absorption of the omega-3 PUFAs and GLA for the emulsion group compared to that observed with the oil group. Particularly noteworthy is the observation that the absorption of EPA and DHA for the non-emulsified oil group was only 33.6% and 44.3% respectively of that observed for the emulsified oil group (Table 3).

**Table 3:** Comparison of AUCs for individual PUFA.

**PUFA**	**Oil group AUC (hr.mg/l)**	**Emulsion group AUC (hr.mg/l)**	**Difference (hr.mg/l)**	**oil/emulsion ratio × 100 (%)**	**95% CI**		**P – value**
						
					**Lower Limit**	**Upper Limit**	
GLA	96.2	139.8	43.5	68.8	0.1	86.9	0.0493
ALA	187.2	284.2	97.0	65.9	22.1	171.9	0.0137
EPA	139.3	414.2	274.9	33.6	175.5	374.3	<0.0001
DHA	97.1	219.4	122.3	44.3	9.2	235.3	0.0355

AUC values are given as Least Square Mean for AUC which is a mean value of total AUC adjusted by the period and sequence.

ANOVA model with repeated measurements taking into account of the cross-over design was used to test for a significant difference in the effect of treatment.

*number of subjects = 22; PUFA – polyunsaturated fatty acid

**Figure 2:**
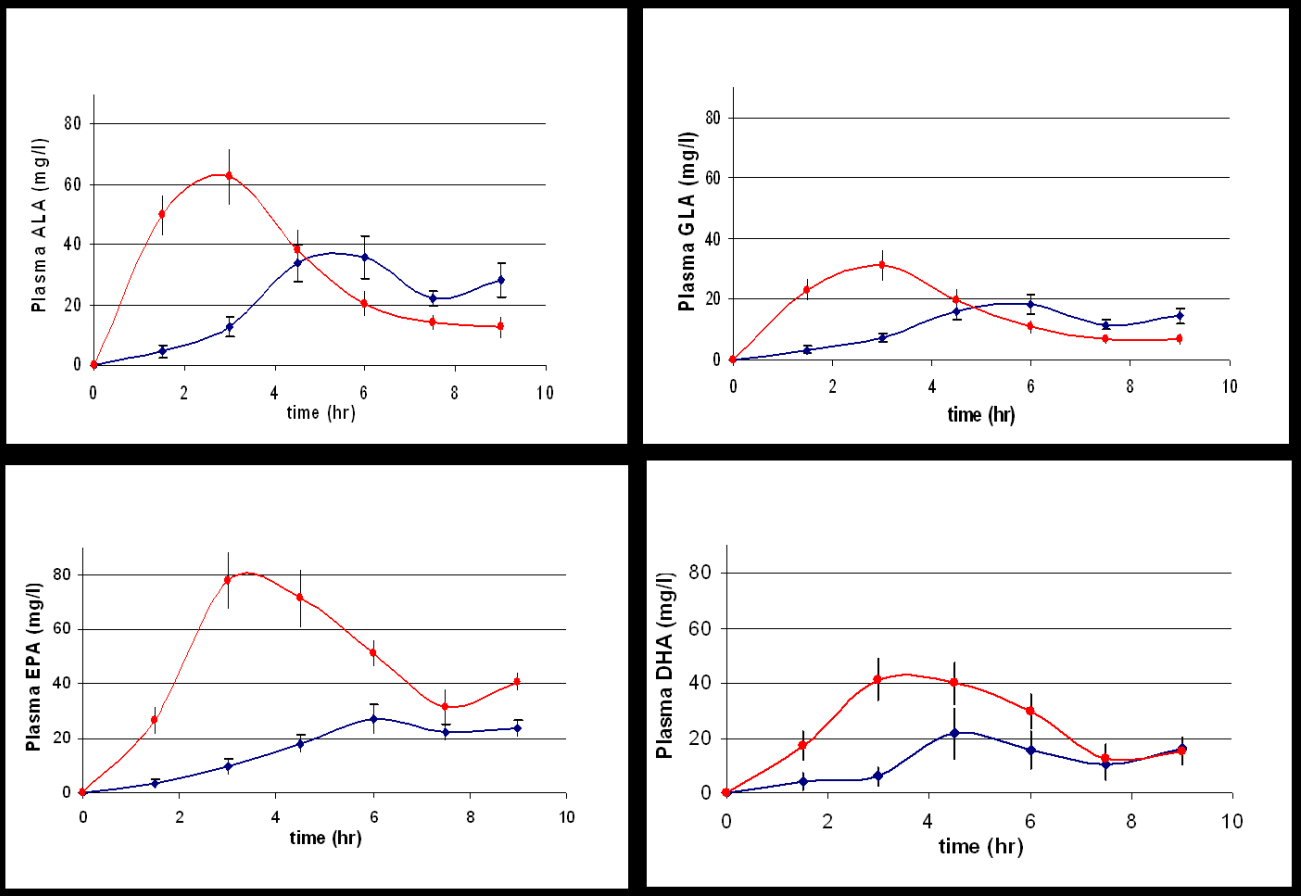
**Comparison of the concentration of plasma polyunsaturated fatty acids for oil and emulsion groups**. Values represent Mean ± SEM of individuals (n = 22) at each time point. -◆- (blue) oil group; -▪- (red) emulsion group. ALA – α Linolenic acid; GLA – γ Linolenic acid; EPA – Eicosapentaenoic acid; DHA – Docosahexaenoic acid. SEM- standard error of the mean; n – number of subjects.

**Figure 3:**
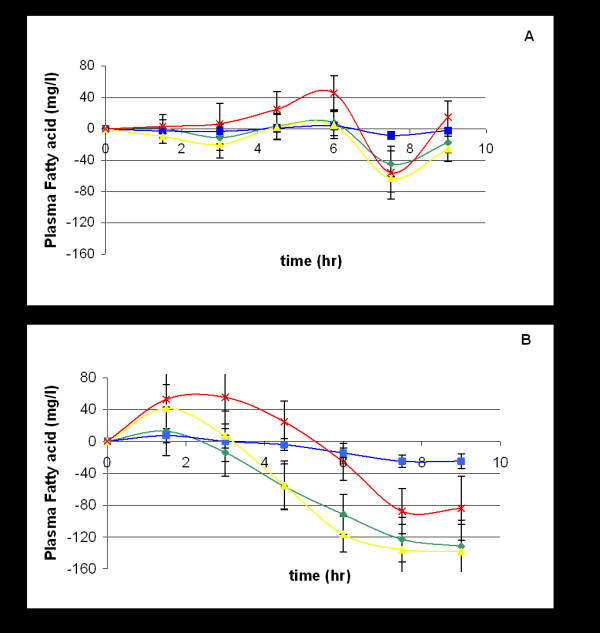
**Plasma fatty acid concentrations for oil (A) and emulsion (B) groups**. Data given as mean for concentration of individuals (n = 22) at each time point. A – oil group; B – emulsion group. -◆- (green) palmitic acid; -▪- (blue) stearic acid; -▲- (yellow) oleic acid; -x- (red) linoleic acid. SEM – standard error of the mean; n – number of subjects.

Comparison of the absorption of the PUFAs for both oil and emulsified oil groups is shown in Figure 2 and it can be seen that the plasma levels of the PUFAs in the emulsion group increased more rapidly than with the oil group in all cases and that increased total absorption of PUFAs, as determined by AUC, was observed for the emulsion group. There were no significant differences between the two groups for the extent of absorption of either the saturated fatty acids (SFA) C16:0 and C18:0, the monounsaturated fatty acid (MUFA) C18:1, n-9 or the PUFA C18:2, n-6 (Figure 3) but there was a trend towards a more rapid postprandial reduction in the plasma concentration of these fatty acids in the emulsion group which reached significance when compared with the oil group with regard to total AUC for palmitic acid (C16:0) P = 0.0319 (95% CI of -864.8 to -43.5).

## Discussion

In this feeding study with a test meal comprising approximately 35% of the calories as fat (either as the mixed oils or the emulsified version of the oils) it has been found that the extent of both the postprandial plasma TAG absorption and also of the individual fatty acids EPA, DHA, ALA, and GLA was significantly higher in the group receiving the emulsified oil mixture compared with the group receiving the non-emulsified form. However, no significant differences were observed for the absorption of the other major dietary fatty acids. These results support previous work from our laboratory which found that the emulsification of fish oil increased both the rate and total postprandial absorption of EPA and DHA (unpublished data).

In a small acute feeding study where the test meal comprised solely fish oils or emulsified fish oils, the absorption of n-3 fatty acids from fish oils was six times greater when presented in an emulsified form, indicating the potential benefits from emulsification [12]. Conversely, no differences were found in a second acute feeding study [14] where the fish oil or emulsified fish oil content of the total fat intake in the test meal (providing approximately 60% of calories as fat) represented about 24% of the total fat content; the remaining 76% of the fat consisted primarily of emulsified dietary fats. Under these conditions there were no significant differences in the absorption of the n-3 fatty acids and the authors concluded that the composition of the total fat content of the test meal had influenced the comparative absorption of the fatty acids.

Our own laboratory work (not shown) suggests that mixing non-emulsified fish oil with a high background level of other emulsified dietary fats confounds the comparison of absorption of emulsified and non-emulsified fish oil.

Several factors have been identified that could contribute a potential for the depressed absorption of omega-3 fatty acids which could be abrogated by pre-emulsification. This includes the nature of the test meal and background diet [17], the influence of droplet size of the oil emulsion [18], the inherent resistance of longer chain fatty acids, especially EPA and DHA, to pancreatic lipase [19] and the delay in the enterocyte re-synthesis of TAG [20].

Chen et al [21] found that there was a significant reduction in the level of fatty acids recovered from the lymph of rats fed a fish oil diet compared with a corn oil diet and Zampelas et al [22] found acute feeding of a fish oil or a plant oil mixture caused an immediate postprandial reduction in plasma TAG levels in the fish oil group. However, Harris et al [17] found that prolonged feeding of a background diet high in fish oil was necessary to cause reduced plasma TAG recovery.

It has been found that lowered pancreatic lipase activity occurs with triglycerides comprising high proportions of EPA and DHA in the sn1 and sn3 positions [7,23] resulting in slower lipolysis of omega-3 fatty acids. The fish oil used in our study had a distribution of EPA and DHA in the sn1 and sn3 positions of 80% and 34% respectively, suggesting that the enzymatic release of EPA in particular may have been retarded. It is interesting that, in this study, the greater absorption of EPA occurred in the emulsified form, and it is tempting to speculate that the effect of emulsification may have been to improve the rate of reaction of pancreatic lipase on the release of EPA and DHA from the sn-1 and sn-3 positions. More research is necessary to confirm this hypothesis.

Ikeda *et al *[20] proposed that inefficiency in the lipolysis of EPA and DHA could delay or lower the transfer of MAGs and FFAs into the enterocyte, limiting the supply/availability of sn2-MAG necessary for the re-synthesis of TAG for incorporation into chylomicrons; this could account for the lower levels of omega-3 fatty acids with the non-emulsified oil group.

However, it is feasible that the rate and extent of absorption of long chain PUFAs may depend as much on the degree of emulsification as on enzymic lipolysis. Borel *et al *[13] showed greater gastric lipase activity with a fine emulsion than with a coarser one and Armand *et al *[18] comparing fine and coarse emulsions of mixed fish and plant oils found 12.7% – 35.6% disappearance of TAG in the stomach with the fine emulsion compared with 4.2–14.8% with the coarse emulsion; for duodenal TAG the disappearance with fine and coarse emulsions was 56.8–73.3% and 34.2–46.3% respectively. This suggests that the efficiency of both the lingual/gastric and pancreatic lipases is improved with the presentation of the oil in a highly emulsified form. The emulsified version of the oil mixture in this study had a median droplet size of 1.3 μm and the profiles for the absorption of the emulsion mixture differ from those for the oil mixture (Figure 3) with more rapid and extensive absorption occurring with the emulsion.

One further possible explanation for the increased levels of plasma EPA and DHA with the emulsions could be the increased contribution from endogenous fatty acids particularly from biliary acids. Whilst this possibility cannot be ignored, studies suggest that the contribution of endogenous EPA and DHA to their total post-prandial levels is minimal [24].

We therefore believe that the lower absorption observed with the oil group could be explained by insufficient emulsification of the oil during the digestion of the oil leading to reduced enzymic lipolysis of the longer chain PUFAs, particularly EPA and DHA. This insufficiency could have led to both a delay and a reduction in the incorporation of these fatty acids into chylomicrons with the consequent reduction in the postprandial TAG concentrations observed. Pre-emulsification of the oil prior to ingestion reduces droplet size and enables improved lipolysis, as reported by Armand *et al *[18], resulting in the significantly improved absorption recorded with the emulsion group. Interestingly, no differences were found between the groups for the digestion and absorption (as determined by AUC) for the SFA, MUFA and PUFA (18:2 6) suggesting that the improved absorption conferred by pre-emulsification is restricted to the longer chain PUFAs, particularly the omega-3 family. This is an important observation because, with typical daily intakes of omega-3 fatty acids being less than 5% of total fat intake, the potential for a pro-atherogenic response based on increased absorption of these particular fatty acids in unlikely.

We observed a trend towards an accelerated reduction in plasma levels of the SFAs, MUFA and PUFA 18:2.6 with the emulsified oil group, which reached significance with palmitic acid suggesting greater clearance from the chylomicrons was occurring. In support of this, Park et al [25] and Zampelas et al [22] have shown that elevated levels of plasma EPA and DHA resulting from acute feeding induce an increase in the activity of lipoprotein lipase (LPL) which is the enzyme responsible for chylomicron TAG degradation and clearance. Moreover, lipoprotein lipase is known to preferentially hydrolyse non omega-3 fatty acids from TAG rich chylomicrons [26].

In our study, the results obtained suggest that the elevated level of EPA and DHA with the postprandial plasma TAG of the emulsified group may have influenced the LPL activity thereby contributing to the accelerated rate of the fatty acid removal observed. The effect of acute feeding of EPA and DHA on LPL has also been observed with the chronic feeding of EPA and DHA resulting in increasing levels of TAG clearance and leading to the clinically relevant observation of omega-3 fatty acids reducing plasma TAG levels [25].

## Conclusion

The study reported here shows that pre-emulsification of an oil mixture increases the absorption of the longer chain more highly unsaturated fatty acids especially EPA and DHA, but not of the shorter chain less unsaturated fatty acids.

As there is now general guidance to the population in the UK and elsewhere to increase the dietary intake of omega – 3 fatty acids, either by increased consumption of oily fish or by use of fish oil supplements, these results suggest that the pre-emulsification of these supplemental fish oils would significantly improve their absorption.

## Competing interests

Obsidian Research Limited provided the funding for this study. Cultech Limited provided the products for the study and received grant support from the Welsh Development Agency to support collaboration with Cardiff School of BioSciences. Dr. Duolao Wang is a statistical consultant to Obsidian Research Limited.

## Authors' contributions

NTP was responsible for the trial design, provision of product and preparation of paper.

SFP, IG and JT were responsible for implementation of the trial, data processing and preparation of the paper.

IAG was responsible for chemical analysis and interpretation of the data.

DW was responsible for statistical processing of the data and interpretation of results.
